# 1883. Features and Outcomes of Tuberculosis in Documented versus Undocumented Migrants in Israel

**DOI:** 10.1093/ofid/ofad500.1711

**Published:** 2023-11-27

**Authors:** Iris Zohar, Adam Pomeranz, Orna Schwartz Harari, Debby Ben-David, Yasmin Maor

**Affiliations:** Wolfson Medical Center, Holon, HaMerkaz, Israel; Wolfson Medical Center, Holon, HaMerkaz, Israel; Wolfson Medical Center, Holon, HaMerkaz, Israel; Wolfson Medical Center, Holon, HaMerkaz, Israel; Wolfson Medical Center, Holon, HaMerkaz, Israel

## Abstract

**Background:**

In countries with low tuberculosis (TB) incidence, most TB cases are observed in foreign-born individuals, including permanent immigrants, and undocumented migrants. There is limited information regarding the differences within these groups. In Israel, where TB prevalence is < 2 cases/100,000 persons, 85% of TB cases occur in foreign-born people. We examined risk factors, clinical course, and outcomes between documented and undocumented immigrants hospitalized with active TB.

**Methods:**

A retrospective analysis of medical records from foreign-born active TB patients admitted to a 700-bed hospital in the center of Israel between January 1st, 2019, to March 31st, 2023. We included patients who initiated TB treatment during hospitalization and were discharged by April 2023. Undocumented migrants were defined as non-Israeli citizens without medical insurance. We summarized their clinical characteristics, course, and outcomes.

**Results:**

48 patients were included, 21 (43.8%) were undocumented migrants. Undocumented migrants were younger (mean age 40.9 vs. 53.0 years, P< 0.001) and predominantly arrived from Africa (95.2% vs. 29.6%, P< 0.001). The prevalence of HIV was similar between both groups. Presenting symptoms, duration of symptoms before admission, rates of bilateral and cavitary pulmonary disease, and extrapulmonary disease were comparable. Multi-drug resistant TB was rare (0% vs. 11.5%, P=0.242). Among patients with positive sputum smears and follow-up, undocumented migrants experienced a longer time to achieve negative sputum cultures (76.0 days vs. 36.8 days, P=0.004), and hospitalizations lasting over 30 days were also more common among undocumented migrants (71.4% vs. 40.7%, P=0.045). Multivariate analysis identified undocumented migrant status, thrombocytosis, and bilateral and cavitary pulmonary disease as risk factors for prolonged hospitalization.

**Conclusion:**

Although the clinical features of TB were similar between documented and undocumented migrants, the response to treatment was slower, and hospital stays were longer among undocumented migrants. This may be due to undocumented migrants presenting with more advanced disease or lack of suitable conditions for isolation and treatment at home.

**Disclosures:**

**Yasmin Maor, MD**, Astra Zeneca: Advisor/Consultant|Astra Zeneca: Honoraria|Gilead: Honoraria|GSK: Honoraria|kamada: Honoraria|Medison: Honoraria|MSD: Advisor/Consultant|MSD: Honoraria|Pfizer: Grant/Research Support|Pfizer: Honoraria

